# Factors associated with and 1-year outcomes of fear of falling in a geriatric post-hip fracture assessment

**DOI:** 10.1007/s40520-022-02159-z

**Published:** 2022-06-21

**Authors:** Roope Jaatinen, Tiina Luukkaala, Markus T. Hongisto, Minna A. Kujala, Maria S. Nuotio

**Affiliations:** 1 Department of Geriatric Medicine, Hospital District of Southern Ostrobothnia, Hanneksenrinne 7, 60220 Seinäjoki, Finland; 2 Oulunkylä Rehabilitation Center, Käskynhaltijantie 5, 00640 Helsinki, Finland; 3 Division of Geriatric Medicine, Department of Clinical Medicine, University of Turku, 20014 Turku, Finland; 4 Research, Development and Innovation Center, Tampere University Hospital, Teiskontie 35, 33521 Tampere, Finland; 5 Health Sciences, Faculty of Social Sciences, Tampere University, 33014 Tampere, Finland; 6 Faculty of Medicine and Health Technology, Tampere University, 33014 Tampere, Finland; 7 Division of Orthopaedics and Traumatology, Hospital District of Southern Ostrobothnia, Hanneksenrinne 7, 60220 Seinäjoki, Finland; 8 Research Services and Department of Clinical Medicine, Turku University Hospital, Kiinamyllynkatu 4-8, 20521 Turku, Finland; 9 Turku University Hospital and University of Turku, Kiinamyllynkatu 4-8, 20521 Turku, Finland; 10 Welfare Division, City of Turku, Turku, Finland

**Keywords:** Fear of falling, Hip fracture, Cognition, Rehabilitation

## Abstract

**Background:**

Hip fracture causes not only physical injury but also psychological trauma. Fear of falling (FoF) is related to poor recovery, loss of mobility and mortality. There is limited data on the clinical factors affecting post-hip fracture FoF and its consequences.

**Objective:**

To investigate the factors associated with and 1-year outcomes of post-hip fracture FoF.

**Methods:**

An observational prospective cohort study. Data were collected on hospital admission, at a geriatric outpatient assessment 4–6 months post-hip fracture and by telephone interviews 1 year after the index fracture. FoF was assessed with a dichotomous single-item question. Logistic regression analyses were conducted to examine the age, gender and multivariable-adjusted association between baseline and the geriatric assessment domains with FoF. Follow-up outcomes included changes in mobility, living arrangements and mortality.

**Results:**

Of the 916 patients included, 425 (49%) had FoF at the time of their geriatric assessment. These patients were predominantly female and were living alone in their own homes with supportive home care. They scored lower on tests of physical performance. Less FoF was documented in patients with diagnosed cognitive disorders before the index fracture and in those with Clinical Dementia Rating ≥ 1. After adjusting for age and gender, no association was observed between FoF and any of the 1-year follow-up outcomes.

**Conclusion:**

Post-hip fracture FoF is common and associated with female gender, polypharmacy, poor daily functioning, poor physical performance and depressive mood. Patients with cognitive disorders have less FoF than those without. FoF appears to have no impact on the follow-up outcomes.

## Introduction

Falls are serious accidents for ageing individuals. Older patients are at high risk for a fall causing hip fracture, which consequently endangers mobility and autonomy [1–3]. The incidence of hip fractures is projected to increase as the population ages worldwide [4, 5]. Mortality is high in hip fracture patients, likewise the risk for institutionalization and need for additional assistance in daily activities [6]. Rehabilitation processes are often strenuous and many patients will not regain their pre-fall functional capacity. Numbers of previously independent patients needing support up to a year post-fracture ranges from 25 up to 75% [7]. Understanding the potentially amendable factors is important to prevent falls and hip fractures, improve rehabilitation outcomes and reduce the burden on health care systems [8].

In addition to physical injury, falling can cause psychological trauma, fear of falling (FoF), even when associated injuries are not functionally limiting [9]. A systematic review by Jørstad and co-workers reported that the psychological consequences related to falling may be even more disabling than the fall itself [10]. The negative impact of falling on quality of life has been reported to be higher than the impact of stroke or cancer [11]. FoF has a bidirectional influence: it is both a risk factor for falls and a consequence of a fall. It has been associated with subsequent poorer quality of life, functional decline, depression and frailty [8, 9]. Thus, FoF may initiate a vicious cycle that reduces participation in activities, impairs rehabilitation outcomes, increases social isolation, provokes new trauma, exacerbates developing deficits and impairs overall prognosis [6, 9, 12, 13].

FoF is common in both fallers and non-fallers [10, 14]. A history of falls, female gender and old age are often reported as major risk factors for developing FoF. In patients with fall-related hip fracture, the prevalence has been reported to be from 21 to 95% [10, 14]. FoF is also related to other psychological factors, such as depression, anxiety and psychological inflexibility [15]. Deandrea and colleagues found in their systematic review that FoF is associated with an approximately three-fold risk of falling [16]. Unfortunately, patients with cognitive impairments are often excluded from studies even though FoF has been suggested as a prodromal symptom of cognitive impairment [17–19].

Previous studies have established that the most significant impact of FoF on health outcomes occurs not immediately, but later in the rehabilitation process, when patients return to their pre-fracture lifestyle [6, 8]. Moreover, FoF seems to have a greater impact on health outcomes in patients with higher baseline functioning [8]. However, comparison of the results of earlier studies is rendered difficult by a variety of methodological issues, such as ambiguity in the definition of FoF, differences in measuring tools, variation in study designs and timing of the assessments etc. [14]. Patients with cognitive disorders are often excluded from trials, thereby removing substantial material from datasets [20]. There is also a gap in the knowledge of the clinical features associated with fear of falling.

The primary aim of our study was to investigate factors associated with post-hip fracture FoF assessed at a comprehensive geriatric assessment (CGA) four to six months after hip fracture in a real-life prospective cohort of older hip fracture patients. The secondary aim was to explore the prognostic significance of post-hip fracture FoF in the change in mobility and living arrangements and mortality up to 1-year post-fracture.

## Methods

All hip fracture patients aged 65 years or more from a geographically defined area with approximately 200,000 inhabitants have been treated in orthogeriatric collaboration in the same hospital since 2007. Briefly, the patients’ acute intervention (emergency treatment and orthopaedic surgery) and postoperative ward care (median length of stay: 6 days, interquartile range, IQR, 6–7 days) took place in comparable circumstances for each patient. Thereafter, the patients were transferred to local health care centre wards for rehabilitation. After discharge from the hospital follow-up telephone interviews were conducted by a geriatric nurse. All patients were invited to an outpatient CGA at the geriatric clinic four to six months post-hip fracture. The treatment pathway designed has been described in detail elsewhere [21, 22]. The study sample for the present study comprised consecutive patients aged 65 years or more suffering their first hip fracture between September 2007 and January 2019 (*n* = 2320). Periprosthetic and pathological fractures were excluded.

Data collection was initiated on admission and continued during the hospital stay. In addition to age and gender, comorbid characteristics were described according to the American Society of Anesthesiologist (ASA) score (ASA 1 = normal healthy patient, ASA 2 = mild systemic disease, ASA 3 = severe systemic disease, ASA 4 = severe systemic disease, constant threat to life, ASA 5 = morbid patient, unlikely to survive) and categorized into two groups: 1–3 and 4–5 [23]. The index accident was recorded as occurring either indoors or outdoors. Known pre-fracture cognitive disorder diagnosed according to the national guidelines by a specialist in neurology or geriatrics was documented [24]. The number of medications in regular use was scrutinized and categorized into three groups: less than four medications, from four to ten medications or above ten medications. Nutritional status was measured using the Mini-Nutritional Assessment-Short Form (MNA-SF), which is a short screening tool for nutritional status with documented clinical relevance and validation in older populations [25]. It contains six questions related to nutritional and health conditions, mobility, and cognition. The MNA-SF score ranges from zero to fourteen. In the present study, categorization was into three groups: normal (12–14), at risk of malnutrition (8–11), or malnourished (0–7). Mobility was categorized as independent or non-independent according to assistance needed. Needing any personal assistance in moving either indoors and/or outdoors was interpreted as non-independent mobility. Living arrangements were categorized as living in own home without organized home care, living in own home with organized care or having assisted living arrangements (e.g. institution) providing 24-h care. Living modality was documented whether living alone (with or without home care) or with company, such as a spouse, a relative, caregiver or in an institution with other residents (Table 1*.*).

**Table 1:** Distribution of baseline characteristics according to having or not a having fear of falling and association of the characteristics with fear of falling (n = 916)

Baseline	Fear of falling						
	Yes (*n* = 452, 49%)	No (*n* = 464, 51%)	*p*	Adjusted for age and gender		Multivariable adjusted	
	*n *(%)	*n *(%)		OR	(95% CI)	OR	(95% CI)
Gender			**0.002**				
Male	106 (24)	151 (33)		1.00		1.00	
Female	346 (76)	313 (67)		**1.52**	**(1.13–2.04)**	**1.46**	**(1.08–1.98)**
Age			0.118				
65–79	143 (32)	177 (38)		1.00		1.00	
80–89	237 (52)	220 (47)		1.26	(0.95–1.68)	1.22	(0.89–1.68)
≥ 90	72 (16)	67 (15)		1.23	(0.82–1.83)	1.02	(0.65–1.58)
ASA score							
1–2	82 (18)	99 (21)	0.368	1.00		1.00	
3	289 (64)	298 (64)		1.01	(0.78–1.55)	0.89	(0.61–1.30)
4–5	69 (15)	55 (12)		1.40	(0.87–2.25)	1.02	(0.61–1.71)
Scene of accident			**0.010**				
Indoors	347 (77)	315 (68)		1.00		1.00	
Outdoors	91 (20)	132 (28)		**0.68**	**(0.49–0.93)**	0.74	(0.52–1.04)
Diagnosed cognitive disorder			**0.033**				
No	352 (78)	333 (72)		1.00		1.00	
Yes	100 (22)	131 (28)		**0.66**	**(0.48–0.89)**	**0.51**	**(0.36–0.73)**
Number of medications			**0.022**				
< 4	69 (15)	104 (22)		1.00		1.00	
4–10	293 (65)	276 (60)		**1.55**	**(1.09–2.19)**	**1.56**	**(1.07–2.26)**
> 10	90 (20)	84 (18)		**1.55**	**(1.01–2.38)**	1.42	(0.88–2.28)
MNA-SF			0.078				
12–14	267 (59)	293 (63)		1.00		1.00	
8–11	169 (37)	159 (34)		1.12	(0.85–1.47)	1.13	(0.83–1.53)
0–7	14 (3)	12 (3)		1.23	(0.56–2.73)	1.06	(0.46–2.43)
Mobility							
Independent	277 (61)	310 (67)	0.217	1.00		1.00	
Non-independent	173 (38)	152 (33)		1.20	(0.90–1.58)	1.27	(0.88–1.84)
Living arrangements			**0.028**				
Home, independent	221 (49)	260 (56)		1.00		1.00	
Home, supported	102 (23)	70 (15)		**1.59**	**(1.10–2.29)**	1.50	(0.95–2.35)
Serviced facilities	45 (10)	45 (10)		1.18	(0.75–1.86)	1.15	(0.69–1.91)
Unknown	84 (19)	89 (19)		1.05	(0.73–1.50)	0.99	(0.61–1.60)
Living modality			0.061				
With company	249 (55)	284 (61)		1.00		1.00	
Alone	203 (45)	180 (39)		1.20	(0.91–1.56)	1.00	(0.68–1.45)

Multivariable model was simultaneously adjusted for all the variables included in the table. ASA = American Society of Anesthesiologists-score, MNA-SF = Mini-Nutritional Assessment, short form. Differences between fear of falling groups were tested using the Pearson chi-square test or Fisher’s exact test, and logistic regression analysis showing results by odds ratios (OR) with 95% confidence intervals (CI). Results for unknown data were shown if results were statistically significant (*p* < 0.05) or nearly significant (0.05 > *p* < 0.10), and if number of unknown data was over 20%. Statistically significant results were expressed in bold

At the outpatient assessment, patients were interviewed to elicit further falls since the hip fracture, whether they suffered from pain in the operated hip or had urinary incontinence defined as any involuntary leakage of urine. Orthostatic blood pressure was measured and defined as positive if blood pressure decreased according to the diagnostic standards (systolic: 20 mmHg or more, or diastolic: 10 mmHg or more respectively) [26]. Fracture types were defined as femoral neck, intertrochanteric or subtrochanteric fractures. Nutritional status was assessed using the MNA-SF by the same methods as before. Basic and instrumental activities of daily living (BADL, IADL) according to Katz and Lawton-Brody respectively [27, 28]. To assess cognition, the Mini-Mental State examination was carried out by experienced geriatric nurses and categorized into normal cognition > 25 and mild 21–25, moderate 12–20 or severe < 12 cognitive dysfunction [29]. In addition, the Clock Drawing Test (CDT) was documented as well as the degree of cognitive decline measured by the Clinical Dementia Rating-score [29, 30]. Depressive symptoms were assessed using the 15-item Geriatric Depression Scale (GDS-15), which has been found to be both a sensitive and a specific screening tool in geriatric settings [31]. A physiotherapist’s assessment preceded the outpatient geriatric assessment. Data on the Timed Up and Go-test (TUG) and Elderly Mobility Scale (EMS) were documented for this study [32, 33]. Categorizations of both measures were according to validated structures. Grip strength was analysed with the Jamar dynamometer and categorized according to the 2019 update on the European Working Group on Sarcopenia in Older People (EWGSOP2) criteria (less than 27 kg in men or less than 16 kg in women) [34] (Table 2).

**Table 2:** Distribution of domains of the outpatient assessment according to having or not having fear of falling (*n* = 916)

Outpatient assessment	Fear of falling						
	Yes (*n* = 452, 49%)	No (*n* = 464, 51%)	*p*	Adjusted for age and gender		Multivariable adjusted	
	*n *(%)	*n *(%)		OR	(95% CI)	OR	(95% CI)
New fall before outpatient assessment			**0.010**				
No	361 (81)	395 (88)		1.00		1.00	
Yes	83 (19)	56 (12)		**1.61**	**(1.11–2.33)**	1.33	(0.89–1.99)
Pain in operated hip			0.291				
No	301 (67)	324 (70)		1.00		1.00	
Yes	147 (33)	136 (30)		1.15	(0.87–1.52)	1.02	(0.75–1.38)
Urinary incontinence			0.057				
No	161 (36)	194 (42)		1.00		1.00	
Yes	285 (64)	265 (58)		1.19	(0.91–1.57)	1.04	(0.74–1.45)
Orthostatic hypotension^a^			**0.034**				
No	318 (70)	347 (75)		1.00		1.00	
Yes	94 (21)	67 (14)		**1.59**	**(1.12–2.26)**	1.42	(0.97–2.07)
Unknown	40 (9)	50 (11)		0.86	(0.55–1.34)	**0.48**	**(0.26–0.88)**
Fracture type			0.154				
Femoral neck fracture	271 (60)	283 (61)		1.00		1.00	
Pertrochanteric fracture	140 (31)	154 (33)		0.92	(0.69–1.23)	0.86	(0.63–1.16)
Subtrochanteric fracture	40 (9)	26 (6)		1.55	(0.92–2.63)	1.54	(0.88–2.72)
MNA-SF			0.088				
12–14	186 (41)	215 (46)		1.00		1.00	
8–11	214 (47)	206 (44)		1.16	(0.88–1.53)	1.00	(0.72–1.38)
≤ 7	50 (11)	41 (9)		1.33	(0.84–2.10)	1.11	(0.64–1.90)
BADL			**0.002**				
No difficulties, 6	129 (29)	183 (39)		1.00		1.00	
Difficulties, ≤ 5	315 (70)	274 (59)		**1.57**	**(1.18–2.09)**	**1.57**	**(1.04–2.36)**
IADL			**0.008**				
No difficulties, 8	57 (13)	94 (20)		1.00		1.00	
Difficulties, ≤ 7	387 (86)	363 (78)		**1.79**	**(1.23–2.60)**	1.38	(0.87–2.17)
MMSE			0.953				
26–30	112 (25)	112 (24)		1.00		1.00	
21–25	121 (27)	121 (26)		0.96	(0.66–1.39)	0.67	(0.44–1.02)
13–20	150 (33)	151 (33)		0.91	(0.63–1.30)	0.67	(0.41–1.11)
≤ 12	56 (12)	64 (14)		0.78	(0.49–1.23)	0.69	(0.34–1.37)
Clock drawing test			0.256				
5–6	97 (22)	121 (26)		1.00		1.00	
2–4	179 (40)	158 (34)		1.31	(0.92–1.87)	1.24	(0.83–1.86)
< 2	144 (32)	150 (32)		1.09	(0.76–1.58)	1.15	(0.68–1.92)
CDR			**0.005**				
0	77 (17)	81 (18)		1.00		1.00	
0.5	136 (30)	97 (21)		**1.51**	**(1.00–2.28)**	1.05	(0.66–1.66)
1–3	138 (31)	183 (39)		0.72	(0.49–1.07)	**0.41**	**(0.24–0.68)**
Unknown	101 (22)	103 (22)		1.12	(0.73–1.70)	0.78	(0.49–1.23)
GDS-15			**0.001**				
0–6	324 (72)	386 (83)		1.00		1.00	
7–15	105 (23)	56 (12)		**2.28**	**(1.59–3.26)**	**1.97**	**(1.32–2.94)**
Unknown	23 (5)	22 (5)		1.19	(0.65–2.17)	2.40	(0.91–6.33)
TUG			**0.001**				
Normal (1–2)	143 (32)	200 (43)		1.00		1.00	
Moderately abnormal (3–4)	200 (44)	188 (41)		**1.46**	**(1.08–1.97)**	1.39	(0.97–1.98)
Markedly abnormal (5)	35 (8)	21 (5)		**2.45**	**(1.36–4.42)**	**3.14**	**(1.49–6.63)**
Unknown	74 (16)	55 (12)		**1.84**	**(1.21–2.78)**	**3.38**	**(1.76–6.47)**
EMS			**0.021**				
> 14	281 (62)	328 (71)		1.00		1.00	
< 14	147 (33)	114 (25)		**1.48**	**(1.10–2.00)**	1.05	(0.69–1.59)
Grip strength decreased^b^			0.469				
No	100 (22)	118 (25)		1.00		1.00	
Yes	330 (73)	322 (69)		1.32	(0.98–1.79)	1.14	(0.81–1.60)
Unknown	22 (5)	24 (5)		1.09	(0.58–2.05)	0.69	(0.30–1.66)
Living modality			0.167				
With company	283 (63)	310 (67)		1.00		1.00	
Alone	166 (37)	150 (33)		1.15	(0.87–1.51)	**1.45**	**(1.05–2.00)**

Multivariable model was simultaneously adjusted for all the variables included in the table. ASA = American Society of Anesthesiologists-score, MNA-SF = Mini-Nutritional Assessment-Short Form, MMSE = Mini-Mental State Examination, CDR = Clinical Dementia Rating, TUG = Timed Up and Go, EMS = Elderly Mobility Scale, BADL = Basic Activities of Daily Living, IADL = Instrumental Activities of Daily Living, GDS-15 = 15-item Geriatric Depression Scale. Differences between fear of falling groups were tested using the Pearson chi-square test or Fisher’s exact test and logistic regression analysis showing results by odds ratios (OR) with 95% confidence intervals (CI). Results for unknown data were shown if results were statistically significant (*p* < 0.05) or nearly significant (0.05 > *p* < 0.10), or if number of unknown data was over 20%. Statistically significant results were expressed in bold

^a^Definition of orthostatic hypotension: Decrease in systolic blood pressure ≥ 20 mmHg or in diastolic blood pressure ≥ 10 mmHg

^b^Grip strength less than 27 kg in men and less than 16 kg in women

Fear of falling was assessed for practicality and feasibility with a dichotomous single-item question (“Do you have a fear of falling?” or “Are you afraid of falling?”, yes/no). The interview was carried out at the outpatient assessment by geriatric nurses together with the patient and his/her escort.

Follow-up data were collected 1-year post-fracture by a geriatric nurse in telephone interviews. Information was obtained from the patient or, if necessary, with support from next of kin, caregiver or care facility personnel. Mobility was defined as more impaired or same/improved, and living arrangements as more supported or same/less supported. Information on mortality was obtained from the Population Register Center and from the medical records. The Population Register Center is a national institution that holds up-to-date information on dates of deaths. This information is automatically updated to patient information systems.

Cross-tabulation of the baseline factors and the outpatient assessment domains according to FoF was analysed. The statistical differences between groups were tested with Pearson’s chi-square test or Fisher’s exact test for categorical variables. Logistic regression analyses with age- and gender-adjusted and multivariable-adjusted models were conducted to examine the association of clinical attributes with post-fracture FoF. The multivariable models were adjusted for new falls before the outpatient assessment, pain in the operated hip, urinary incontinence, orthostatic hypotension, fracture type, nutritional status, functional and cognitive abilities, depressive mood, physical performance and living modality. The impact of FoF on the 1-year follow-up outcomes was analysed by an age- and gender-adjusted logistic regression analysis for change in mobility or living arrangements. Results of the logistic regression analyses are shown using odds ratios (OR) and 95% confidence intervals (CI). One-year mortality was modelled using age- and gender-adjusted Cox proportional hazard regression analyses. Results are shown as hazard ratios (HR) with 95% CI. Risk-scoring for risk of having FoF was defined by calculating the sum of statistically significant or nearly significant (*p* < 0.10) variables in the multivariable-adjusted logistic regression analyses (female gender, having a cognitive disorder diagnosed pre-fracture, at least four medications, orthostatic hypotension, BADL ≤ 5, IADL ≤ 7, GDS-15 7-15, abnormal TUG and/or living alone). Each of these factors contributed one point. The theoretical maximum was nine points. IBM SPSS Statistics version 26.0 (SPSS Inc. Chicago, Illinois, USA) was used for statistical analyses. *p* values under 0.05 were considered statistically significant.

The study design was approved by the Ethics Committee of the Hospital District of Southern Ostrobothnia. All participants or their representatives gave informed consent.

## Results

**Fig. 1:**
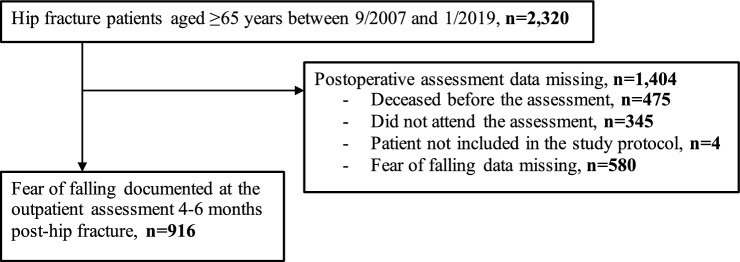
Flow chart of the study population

From the sample including 2,320 patients 475 (20%) had died before the outpatient assessment. A total of 345 (15%) patients had not visited the outpatient clinic within the desired time period. Data on FoF were missing from 580 (25%) patients. Ultimately, 916 (39%) patients had complete documentation of the necessary variables and measures from the geriatric assessment. The flow chart of data collection is presented in Fig. 1*.*

Baseline characteristics according to FoF are presented in Table 1. FoF was more common in women (OR 1.46, 95% CI 1.08–1.98) and in patients using four to ten medications regularly (OR 1.56, 95% CI 1.07–2.2.26). Cognitive disorder diagnosed pre-fracture remained inversely associated with FoF (OR 0.51, 95% CI 0.36–0.73) in the multivariable-adjusted model.

Distribution of the domains of the outpatient CGA according to FoF is presented in Table 2. In the age and gender-adjusted analyses orthostatic hypotension (OR 1.59, 95% CI 1.12–2.26), difficulties in daily activities (BADL OR 1.57, 95% CI 1.18–2.09 and IADL OR 1.79, 95% CI 1.23–2.60), CDR-score of 0.5 (OR 1.51, 95% CI 1.00–2.28), depressive mood (OR 2.28, 95% CI 1.59–3.26) and poorer physical performance were more common in patients with FoF than in those without. In the multivariable model including all variables, EMS lost its significance. FoF was more common in patients living alone (OR 1.45, 95% CI 1.05–2.00) than in those living with a company. Patients with mild to severe dementia as indicated by the CDR score reported less FoF than those with normal cognition or only mild dementia. However, different levels of cognition as measured with the MMSE, or the CDT was not associated with FoF.

**Fig. 2:**
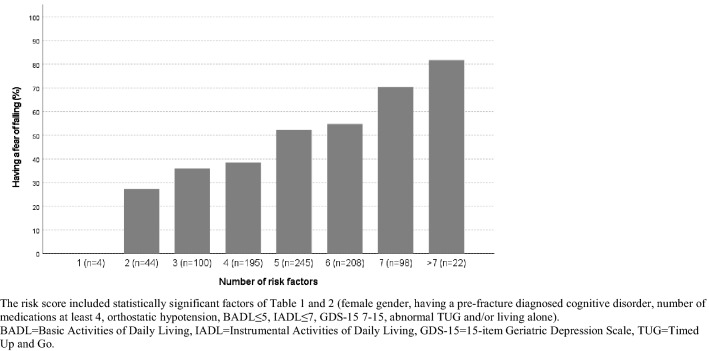
Prevalence (%) of having fear of falling according to number of risk factors. The risk score included statistically significant factors of Table 1 and 2 (female gender, having a pre-fracture diagnosed cognitive disorder, number of medications at least 4, orthostatic hypotension, BADL ≤ 5, IADL ≤ 7, GDS-15 7-15, abnormal TUG and/or living alone). *BADL* Basic Activities of Daily Living, *IADL* Instrumental Activities of Daily Living, *GDS-15* 15-item Geriatric Depression Scale, *TUG* Timed Up and Go

The overall rate of having FoF at the geriatric assessment grew as the number of concurrent risk factors increased (Fig. 2).

Fear of falling was not significantly associated with the follow-up outcomes between outpatient assessment and 1-year post-fracture after adjusting for age and gender: change in living arrangements OR 0.81, 95% CI 0.53–1.23, change in mobility OR 1.15, 95% CI 0.75–1.76, and mortality hazard ratio (HR) 1.25, 95% CI 0.66–2.37.

## Discussion

This study demonstrated the multifactorial nature of post-hip fracture FoF in older hip fracture patients. Patients suffering from FoF were predominantly female, had multiple medications in regular use and were more likely to be living in their own home with supportive home care than were patients without FoF. Patients who had fallen outdoors or patients with cognitive disorders diagnosed pre-fracture demonstrated less post-fracture FoF. Patients with FoF were more likely to have fallen again before the geriatric outpatient assessment than those without FoF. Moreover, they were more likely to have orthostatic hypotension, difficulties in daily activities, depressive mood and they performed poorer in the tests of physical performance. FoF was less likely in patients from whom orthostatic blood pressure could not be obtained and who had a CDR score of 1 or more. Many of the significant predictors in age and gender-adjusted models became irrelevant after adjusting for other clinical parameters, indicating the complex nature of fear in this patient population. The rate of having post-hip fracture FoF increased as the risk factors derived from the multivariable models increased.

The multifaceted nature of FoF has been addressed in a study by Painter and co-workers. Female gender, multiple medications (≥ 4), fall history and difficulties in ADL have been identified as factors contributing FoF. In addition, the decline in physical performance affects the development of post-hip fracture FoF [12]. Our results concur with these findings. In agreement with previous studies, the female gender was also significantly associated with FoF [35]. However, in our data older age was not associated with FoF, contrary to some previous studies [14]. The prevalence of FoF in our data (49%) is in line with earlier studies assessing FoF with a single-item instrument [35–37].

No fundamental definition of FoF has been presented, thus considerable variation in prevalence has been reported. The terms “fear of falling”, “fall efficacy”, “balance confidence” etc. may be used interchangeably to refer to the same condition [38–40]. Earlier investigations suggest that the definition of FoF in each study should be appropriately specified [17]. Measurement instruments should be selected to suit the study design and be feasible in the target patient group [17, 39, 41]. Little is known about comparisons between the various assessment instruments for FoF [10, 14]. A comprehensive systematic review by Visschedjik and co-workers observed that a multicomponent instrument may include details that are irrelevant for frail older patients who are primarily unable to perform some of the measured activities [17]. A review by Jung splits the term FoF into two categories: one that focuses on the fear itself and the other that reflects the loss of confidence while doing a certain daily maneuver [39]. In a review by Scheffer and colleagues, 15 different instruments were compiled, seven to measure self-efficacy and eight to measure FoF [14]. Complex instruments may have a solid theoretical background and they provide more information about the activities that are the cause of the fear but they may not yield reliable information on cognitively impaired patients [40, 42]. Single-item instruments may be more influenced by psychological factors whereas the more elaborate instruments may focus more on physical performance [15]. A single-item instrument may be quick and easy to use but the evidence for its validity is weak [10]. Moreover, a dichotomous answer does not indicate the level of fear [37].

An interesting observation in our study was that patients with a known pre-fracture cognitive disorder were less likely to report FoF than those without cognitive impairments. This finding concurs with the existing literature: patients with cognitive impairment may exhibit “anosognosia”, in other words symptom unawareness, and therefore be incapable of acknowledging functional deficits or FoF [43, 44]. The significant association of CDR score of 1 or more with FoF in the multivariable analysis supports this hypothesis. In fact, patients with milder cognitive impairment (CDR score less than 1) may still be active and enjoy a good mobility level. They probably acknowledge the difficulties in balance control and emerging cognitive difficulties, and therefore, are able to be afraid of falling. Moreover, with higher CDR score, the cognitive capability to verbally express self-efficacy may be compromised leading to underreporting of fear. Conversely, hip fracture patients who fell outdoors were more likely to have better daily functioning, higher mobility level and better cognitive status than those who fell indoors. We hypothesize that these individuals reported less FoF since they were more aware of their own capability and more likely able to participate in rehabilitation activities.

FoF remained strongly and consistently associated with depressive mood measured by the GDS-15 across the analyses while the association of having orthostatic hypotension was reduced after simultaneously adjusting for the other domains of the outpatient CGA. This finding indicates the psychometric value of FoF. Fear is not directly explained by the somatic issues but is more often present together with mental issues, such as depressive symptoms. Depression has been acknowledged as a risk factor for developing FoF and also as a repercussion of FoF [14, 45]. A study by Delbaere and colleagues found that the effect of FoF on functional outcomes 1-year post-fracture may depend on whether the fear is comparable to the actual physiological risk of falls. They found that FoF is strongly associated with psychological factors, such as depressive symptoms [46].

A strength of this study is the substantial patient sample derived from the same prospective cohort of older hip fracture patients. Patients followed our systematic care pathway including standardized measurements. The attendance rate at the geriatric outpatient assessment was high, as reported in our earlier article [47]. Moreover, since all the patients were invited regardless of their health or cognitive status, living arrangements or mobility etc., the results represent the real-life effect of post-hip fracture FoF well on the chosen outcomes.

Several limitations must be conceded. First, a considerable amount of missing data of FoF (25%) must be noted. This may affect the robustness of the prevalence figures of our data. Secondly, we only assessed FoF once during follow-up. Fear is a dynamic phenomenon which may evolve during rehabilitation manifesting as a mild inconvenience to a severely debilitating symptom [17, 48]. We hypothesized that the sense of fear documented at the outpatient geriatric assessment four to six months after the index fracture would be persistent and thus also affect the 1-year outcomes. The trajectory of FoF during rehabilitation deserves more attention in future studies. Thirdly, we used a direct dichotomous question to assess FoF, which may not have the theoretical background of the more complex measures or may not assess the same construct constantly due to the variety of interpretations of fear (“worry”, “concern” etc.). A single-item question may also be affected by the patient’s behaviour or mood at the time of the inquiry, thereby compromising reliability [10, 39]. We have reported in our earlier work that undiagnosed cognitive disorders are common in older hip fracture patients and that an older patient is at high risk of developing cognitive disorders after the hip fracture [21, 47]. Since our patient sample included patients with various cognitive capabilities or compromised abilities in daily activities, more elaborate instruments, such as the Falls Efficacy Scale (FES), the Activity-related Balance Confidence (ABC) scale or the Survey of Activities and FoF among Elderly (SAFFE) were considered too imprecise for our study design. The assessment method was chosen prioritizing the comprehensibility, response rate and reliability of answers.

## Conclusion

Post-hip fracture FoF is common: almost every second older patient experiences fear four to six months after hip fracture. It is more common in women, in patients living alone, and in patients with multiple medications in regular use, difficulties in daily activities or impaired physical performance. Patients with pre-fracture cognitive disorders and those with mild to severe dementia at the outpatient assessment reported less FoF than did to those with better cognitive capability. Decline in mobility, change to more supported living arrangements or mortality at 1-year post-fracture are explained by other factors than FoF itself. Our findings deserve attention in clinical practice. FoF should be identified even though it may not appear in investigations. It may affect rehabilitation but does not solely explain poor outcomes. A CGA is warranted as a benchmark for a rehabilitation assessment.
